# Preparation and Characterization of the Extracellular Domain of Human Sid-1

**DOI:** 10.1371/journal.pone.0033607

**Published:** 2012-04-11

**Authors:** Ashley J. Pratt, Robert P. Rambo, Pick-Wei Lau, Ian J. MacRae

**Affiliations:** 1 Department of Molecular Biology, The Scripps Research Institute, La Jolla, California, United States of America; 2 Life Sciences Division, Lawrence Berkeley National Laboratory, Berkeley, California, United States of America; Nagoya University, Japan

## Abstract

In *C. elegans*, the cell surface protein Sid-1 imports extracellular dsRNA into the cytosol of most non-neuronal cells, enabling systemic spread of RNA interference (RNAi) throughout the worm. Sid-1 homologs are found in many other animals, although for most a function for the protein has not yet been established. Sid-1 proteins are composed of an N-terminal extracellular domain (ECD) followed by 9–12 predicted transmembrane regions. We developed a baculovirus system to express and purify the ECD of the human Sid-1 protein SidT1. Recombinant SidT1 ECD is glycosylated and spontaneously assembles into a stable and discrete tetrameric structure. Electron microscopy (EM) and small angle x-ray scattering (SAXS) studies reveal that the SidT1 ECD tetramer is a compact, puck-shaped globular particle, which we hypothesize may control access of dsRNA to the transmembrane pore. These characterizations provide inroads towards understanding the mechanism of this unique RNA transport system from structural prospective.

## Introduction

In the nematode *C. elegans*, gene-silencing via the RNAi pathway can spread from cell to cell, throughout most of the body of the animal. A genetic screen for mutants deficient in this systemic spread of RNAi identified the gene *sid-1* (systemic interference defective; also termed *rsd-8*) [1], [2]. The encoded protein, Sid-1, is a transmembrane channel required for importing silencing dsRNA into living worms [1] When expressed in cultured *Drosophila* cells (which lack a *sid-1* homolog), the protein promotes a rapid, dose-dependent dsRNA uptake in an ATP-independent [3] and dsRNA-gated [4] manner. Strikingly, Sid-1 is capable of efficiently transporting dsRNA of a large size range (from 21 bp to >500 bp). The internalized dsRNA is competent for gene silencing [3], [5], and RNA retention is dependent on components of the RNA-induced silencing complex (RISC) [4]. Sid-1 is expressed in most non-neuronal cells in the worm, and when ectopically expressed in neurons, can enhance dsRNA delivery to these cells as well [6]. *C. elegans* Sid-1 exogenously expressed in mammalian cells was also shown to enhance cellular dsRNA import and silencing [7]. Although the systemic RNAi response in *C. elegans* is complex [1], [2], involving additional transport factors [8] as well as Sid-1-independent RNAi export mechanisms [9], *C. elegans* Sid-1 is competent and, in some instances, sufficient for importing silencing dsRNA into the cell.

Sid-1 homologs are present throughout the metazoan kingdom. The physiological function of the protein in most animals remains unexplored, but in several cases Sid-1 expression patterns suggest a role in systemic RNAi. For example, in honeybees exposed to dsRNA, expression of Sid-1 increased and peaked just before maximal systemic silencing was observed [10]. Similarly, in the pacific white shrimp, *Litopenaeus vannamei*, Sid-1 expression increased in response to >50 bp dsRNA injection and protected the organism from viral infection [11]. Interestingly though, knock down of three distinct Sid-1-like (*sil*) homologs (individually or in combination) in the beetle *Tribolium castaneum*, did not block systemic RNAi, suggesting the existence of a Sid-1 independent transport system ([12] and see [13]). Indeed, the fruit fly *Drosophila melanogaster* is able to mount a systemic RNAi response when infected with dsRNA viruses even though flies lack a recognizable Sid-1 homolog [14], and both the fly and *C. elegans* can take up dsRNA via endocytosis mechanisms [2], [15], [16].

Many vertebrates have two Sid-1 homologs, SidT1 and SidT2, and evidence exists suggesting that the vertebrate homologs also mediate dsRNA transport. For example, over-expression of human SidT1 enabled uptake of dsRNA into cultured human cells [17]. Likewise, over-expression of SidT2 from the fish *Siniperca chuatsi* in fathead minnow epithelial cells increased the uptake of exogenous dsRNA and also helped protect against viral infection [18]. Moreover, uptake of lipid-conjugated small interfering RNAs (siRNAs) into HepG2 cells was blocked by SidT1 knockdown or by treatment with a SidT1 neutralizing antibody. However, the mechanism of uptake is unclear and the physiological role of SidT1 in mammalian biology is not known. Human SidT1 is expressed predominantly in lymphocytes and dendritic cells [19]. Human SidT2 is more ubiquitously expressed [19], and interestingly, mouse SidT2 was shown to localize predominantly in lysosomes [20]. Therefore, compared to *C. elegans* Sid-1, the functions of the Sid-1 homologs in vertebrates may be more diverse and are certainly less well understood.

Sequence analysis and biochemical experiments suggest that Sid-1 proteins are composed of a large N-terminal extracellular domain and 9–12 transmembrane segments, with an intervening cytosolic loop between segments 1 and 2 [3]. Dominant negative effects of Sid-1 mutants in over-expression experiments suggest the protein functions as an oligomer [5]. Beyond these basic characterizations and predictions very little is known about the structure of the protein and tools for probing the protein's structure are still being developed. Here we show that the extracellular domain of the human homolog SidT1 (SidT1 ECD) can be expressed and purified using a baculovirus system. The purified recombinant protein is glycosylated and spontaneously forms a defined oligomeric structure, most consistent with a tetramer. EM and SAXS measurements suggest that the SidT1 ECD oligomerizes into a compact, puck-shaped globular particle. This study is the first structural characterization of the Sid-1 ECD and paves way for understanding the mechanism and function of the Sid-1 family of proteins.

## Results

### Recombinant SidT1 ECD folds into a stable soluble structure

All Sid-1 homologs have a ∼300 amino acid N-terminal ECD, which is predicted to form a globular, soluble domain with defined secondary structure (Figure S1, discussed below). To test this prediction directly we attempted to produce isolated forms of the Sid-1 ECD. Initial efforts focused on expressing ECDs of Sid-1 homologs as C-terminal fusions to the maltose-binding protein (MBP) in *E. coli*. ECDs of Sid-1 homologs from humans (AAI17223 and AAI14523), *C. elegans* (NP_504372), *Branchiostoma floridae* (XP_002597180), and *Strongylocentrotus purpuratus* (XP_789210) were tested. Although we could produce milligram quantities of each protein, folding defects were always apparent – the proteins co-purified with the chaperone GroEL and eluted in the void volume of size exclusion columns, suggesting that they had formed large, soluble aggregates. Moreover, removal of the MBP tag led to rapid degradation of the purified Sid-1 ECD polypeptides (data not shown). These observations led us to postulate that either Sid-1 ECDs do not form globular structures outside of the full-length protein, or that endogenous Sid-1 ECDs might have posttranslational modifications, absent in bacterial expression systems, that are required for proper protein folding. To explore these possibilities we established a baculovirus system for expression of secreted Sid-1 ECD proteins in insect cells.

We constructed a modified version of pFastBac HT A for the expression of secreted proteins under control of the *Autographa californica* multicapsid nuclear polyhedrosis virus GP64 promoter. The construct included an N-terminal His_6_-tag to be used for protein detection and purification. Our major efforts focused on characterization of the ECD of human SidT1 (isoform 1 of Sid-1 in humans). Baculoviruses encoding the SidT1 ECD were produced in Sf9 cells, and then used to infect either Sf9 or Tni cells. After 1–3 days of infection, the medium was cleared of cells and large debris by centrifugation and filtration and assessed for the presence of soluble SidT1 ECD.

To assay for Sid-1 ECD in the culture meduim we performed small-scale fractionation of the medium by Ni-affinity chromatography. We noticed, however, that the insect cell medium we used contains metal-chelating compounds that cause leaching of Ni^2+^ and Co^2+^ from metal-affinity resins and interfere with the ability of the resins to bind His_6_-tagged proteins. The interfering activity could be removed by dialyzing the medium against Ni-affinity wash buffer. The post-infection medium contained a soluble protein that could be purified by Ni-affinity chromatography and, judging from SDS PAGE, was the approximate molecular weight of the SidT1 ECD. Anti-His_6_ Western blots also revealed the presence of a soluble protein with the approximate size of the SidT1 ECD in the medium 24–48 hours post infection, and tandem liquid chromatography mass spectrometry (MS) identified the isolated protein as SidT1 (data not shown). Therefore, recombinant SidT1 ECD can fold into a soluble form that is stable enough to accumulate to significant levels (>1 mg/L) in cell culture medium.

We next devised a protocol for large-scale preparation of SidT1 ECD for biochemical and biophysical studies. An expression time course revealed that 48 hours was optimal for expression of SidT1 in Sf9 cells and 24 hours was optimal in Tni cells (data not shown). For large-scale preparations, dialysis of the clarified medium was an impractical method for removing the metal-chelating activity. As an alternative, we passed the medium through a fast-flow cation exchange column, which served to concentrate the protein and remove most of the metal-chelating activity. Eluted protein samples were then subjected to Ni-affinity purification, His-tag removal (using TEV protease), and size exclusion chromatography. This procedure generated 0.5–1 mg of ∼99% pure SidT1 ECD protein per liter of cultured insect cells. Due to favorable yield and expression kinetics, we usually produced SidT1 ECD samples in Tni cells. However, Sf9 cells have the benefit of producing PNGaseF-sensitive glycoproteins [21], which were useful in deglycosylation studies (see below).

### Post-translational modification of SidT1 ECD in insect cells

**Figure 1:**
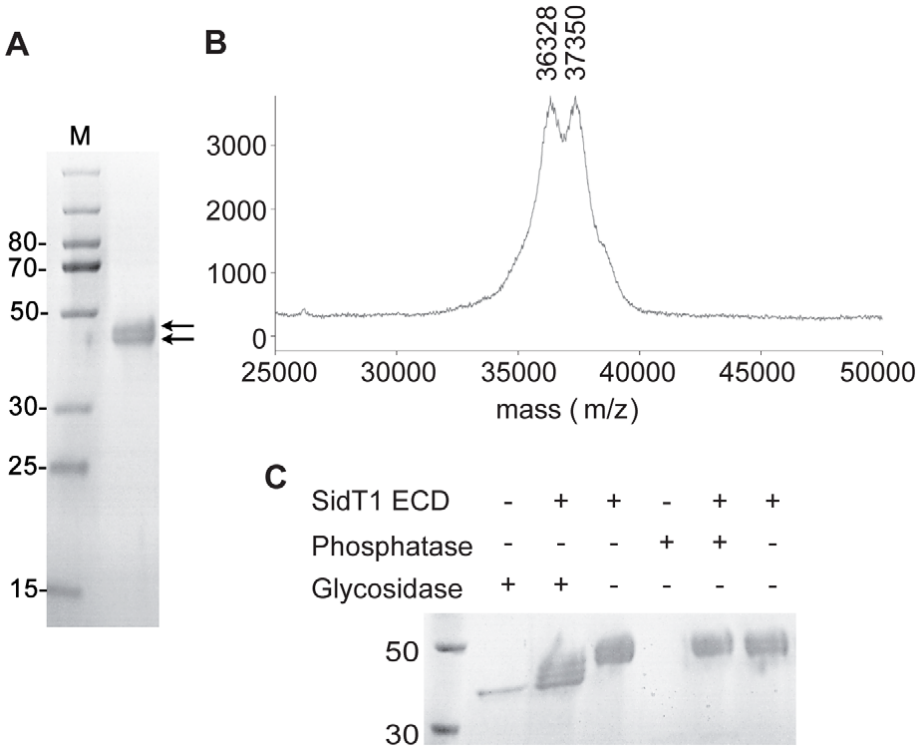
Recombinant SidT1 ECD is a glycoprotein. **A**, Heterogeneity in purified SidT1 ECD samples. Double arrows indicate the presence of two bands on SDS-PAGE. **B**, MALDI-TOF mass spectrum reveals a mixture of two major species of 36.3 and 37.4 kDa. **C**, N-Glycosidase F treatment of the protein resulted in increased electrophoretic mobility, suggesting N-linked glycosylation. Lambda phosphatase treatment had no noticeable effect.

Purified SidT1 ECD migrated as two species on SDS-PAGE, both of which were higher than 34 kDa, the predicted molecular weight of the protein (Figure 1A). Nonetheless, both species were separately identified as SidT1 using tandem liquid chromatography mass spectrometry (data not shown). Likewise, MALDI-TOF MS identified at least two species of 36.3 and 37.4 kDa (Figure 1B). Native SidT1 is presumably folded in the endoplasmic reticulum, where it would be susceptible to post-translational modifications, such as N-terminal glycosylation. The protein also contains potential phosphorylation sites [22]. To assay for the presences of these two types of modification in our recombinant samples we treated purified SidT1 with either lambda phosphatase or N-Glycosidase F (PNGaseF) enzymes. Treatment with phosphatase had no visible effect on the electrophoretic mobility of the protein on SDS PAGE. In contrast, glycosidase treatment caused a substantial increase in electrophoretic mobility (Figure 1C), suggesting the SidT1 ECD produced in insect cells is N-glycosylated. Glycoslyation is likely to be essential for proper protein folding as we noted dramatic reductions in yield when expressing glycosylation site mutants or adding the glycosylation inhibitors kifunensine, swainsonine or tunicamycin to the growth medium during viral infection. We were also unable to detect any SidT1 ECD when expression was directed to the cytoplasm of insect cells.

### SidT1 ECD spontaneously assembles into a stable tetrameric structure

**Figure 2:**
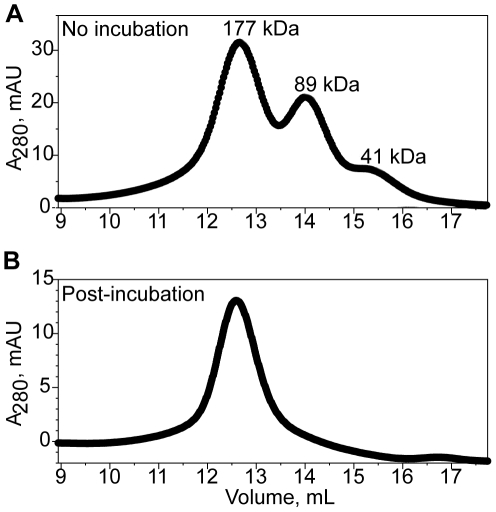
Mature SidT1 ECD forms an oligomer. Size exclusion elution profiles from a Superdex 200 column are displayed. **A**, Concentrated protein applied directly to the column, with no pre-incubation period, elutes as three species with sizes consistent with monomeric, dimeric and tetrameric forms of the protein. **B**, Following an incubation period of two hours, the protein elutes as a homogeneous tetrameric species.

Functional characterization of a dominant negative mutant of *C. elegans* Sid-1 led to the proposal that the full-length protein functions as an oligomer [5]. Interestingly, we also observed evidence for oligomerization of the SidT1 ECD. During the final stages of purification, the protein often eluted as three distinct peaks from the size exclusion column (Figure 2A). Comparing to elution volumes of molecular weight standards, we estimate that the three peaks correspond to particles with molecular masses of 41 kDa, 89 kDa and 177 kDa. The ratio of these masses is thus 0.9∶2∶4, which is most consistent with monomeric, dimeric and tetrameric forms of the SidT1 ECD [although because the estimated mass of the highest molecular weight species is 1.2× greater than 148 kDa, the calculated mass of a tetramer based on MALDI-TOF of the denatured form of the protein (∼37 kDa) we cannot rule out the possibility of a pentamer with certainty]. Re-injection of the fractions containing the putative tetramer did not result in a redistribution of species to the dimeric and monomeric forms, revealing that three oligomeric species are not in rapid equilibrium with each other (data not shown). Furthermore, when the concentrated protein was incubated on ice for several hours before injecting into the column, only the largest species was observed, suggesting that the tetramer is the final, most stable form of the protein (Figure 2B). We also noted that treatment of SidT1 ECD with PNGaseF did not affect the oligomeric state of the protein (Figure S2). Therefore, we hypothesize recombinant SidT1 ECD assembles into a stable and discrete tetrameric structure.

### The SidT1 ECD tetramer is a compact, disc-shaped particle

**Figure 3:**
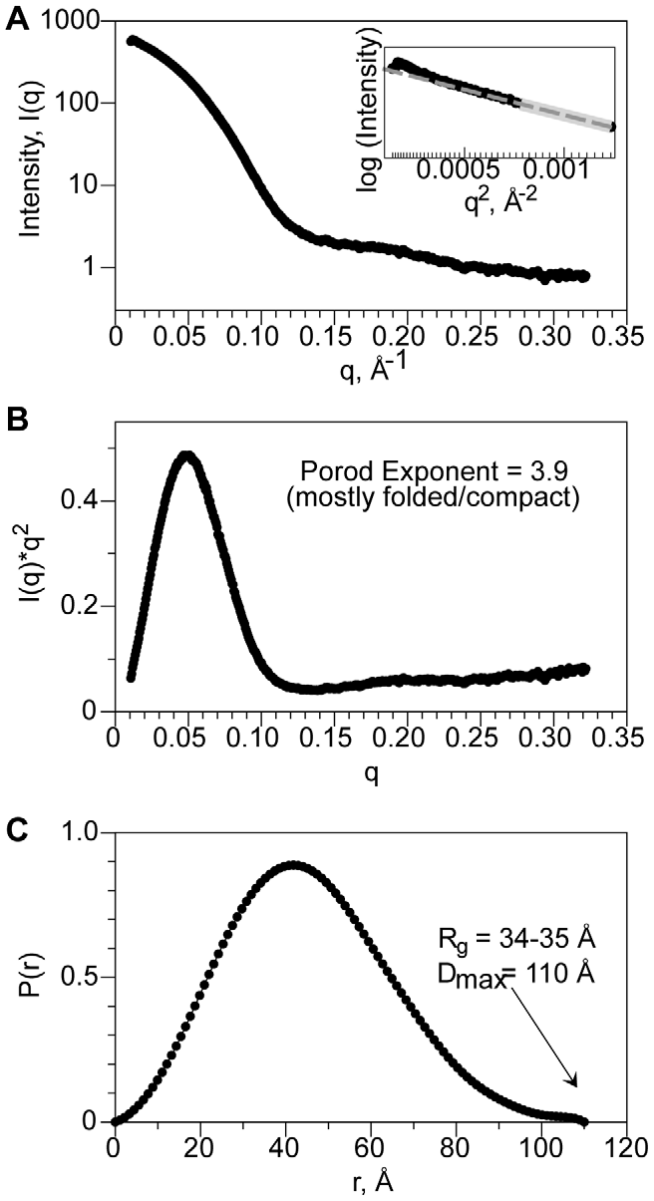
SidT1 SAXS analysis. **A**, Solution SAXS profile for glycosylated SidT1 ECD. Inset displays Guiner region (data <q = 0.028, beyond light gray line, were omitted in subsequent modeling). **B**, P(r) plot of SidT1 ECD reveals a unimodal curve, consistent with a globular protein species. The maximum particle diameter, D_max_, is 110 Å. **C**, Kratky plot of SAXS data shows very little flexibility/looseness, indicated by the convergence of the curve. The Porod exponent is 3.9.

To test our hypothesis that SidT1 ECD forms stable tetramers in solution, we further characterized these particles using small angle X-ray scattering (SAXS). SAXS determines the basic parameters describing the size and shape of the particle (Figure 3A, B, C) [23], [24], revealing that SidT1 ECD has: a radius-of-gyration of approximately 35 Å, a maximal dimension of ∼110 Å and a molecular weight in the range of 160–165 kDa, using the SAXS MoW server [25] (see Table S1). The SidT1 ECD also appears to be a compact and relatively rigid molecule – unlike flexible polymers, which display a 1/q^2^ scattering dependency, the SidT1 ECD data exhibits a 1/q^4^ dependency (Porod exponent = 3.9), indicative of a well-folded, compact particle [26]. The parabolic shape of the Kratky plot is also characteristic of foldedness and minimal flexibility (Figure 3C).

**Figure 4:**
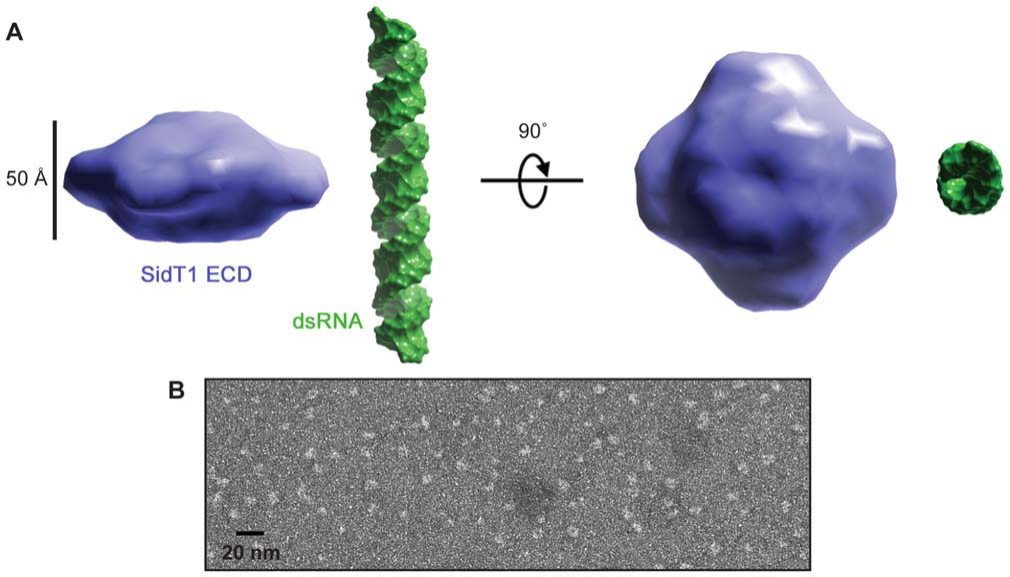
3D model of the SidT1 ECD. **A**, Averaged 3D reconstruction of SAXS data using Gasbor software and P4 symmetry reveals a puck-like shape. **B**, Transmission electron micrograph showing negatively-stained SidT1 ECD particles.

Imposing P4 symmetry, we used the SAXS data to calculate an *ab initio* 3D model of the SidT1 ECD. The resulting model is a disc-shaped particle (Figure. 4A). Importantly, the SAXS structure is consistent with electron micrographs of negatively stained SidT1 ECD, which contained particles of similar shape and dimension (Figure 4B). Calculating models with P3 or P5 symmetry also resulted in disc shaped particles; however these models did not fit the data as well as P4 (Figure S5). The P3 models were less reproducible, with a mean normalized spatial discrepancy (NSD) value significantly greater than that of the P4 models (1.40 versus 1.05, respectively). The data did also not fit the P5 model as well as the P4 model (the average X^2^ was 2.6 and 1.8 for P5 and P4, respectively). Furthermore, based on the scattering data, the estimated protein density for a pentamer would be >1.4 g/cm^2^, which is higher than commonly observed [26]. Therefore, these analyses further suggest that the SidT1 ECD is a tetrameric particle in solution.

We also modeled our data SAXS data using P222 symmetry. The resulting averaged model is an oblate spheroid shape that is similar to the P4 model, though slightly more elongated in one direction perpendicular to the P4 symmetry axis (Figure S5). P222 and P4 fit the data equivalently well (X^2^ is 1.8 and 1.7 for P222 and P4, respectively) and it is not possible for us to distinguish between these two symmetries with the present analysis. However, the overall results reveal that SidT1 ECD forms a well-folded, compact, disc-like tetrameric glycoprotein ∼110 Å in maximal diameter. The non-spherical shape of the particle explains the erroneously high native molecular weight estimated by size-exclusion chromatography (Figure 2A).

## Discussion

This work describes the first preparation of any domain of a Sid-1 protein. We found SidT1 ECD folds efficiently in a eukaryotic secretory pathway and is secreted in a soluble form. We were unable to detect SidT1 ECD when expression was targeted to the cytoplasm of insect cells (by removal of the signal sequence) and noted diminished expression levels when glycosylation of the protein was affected, either by expressing N-linked glycan site mutants or using glycosylation inhibitors (data not shown). We therefore suspect that glycosylation is important for folding and/or stability of the protein. We also note, however, that the full extent of glycosylation present is not required for stability of the mature SidT1 ECD tetramer as partial degylcosylation did not substantially affect the native molecular weight of the oligomer (Figure S2). Recombinant forms of ECDs from human SidT2 (Figure S3) and *C. elegans* Sid-1 (Figure S3, S4) also appear to be glycosylated. Endogenous mouse SidT2 from liver tissue extracts was recently found to contain significant glycosylation [20] – thus, this post-translational modification appears to be a common feature of Sid-1 proteins.

The isolated SidT1 ECD is remarkably stable: The crude, secreted protein accumulated in cell culture medium incubated at 27°C, and, the final, purified sample remained completely intact after a 1-week incubation at room temperature (data not shown). During many preparations we observed what appeared to be monomeric, dimeric and tetrameric forms of the protein. Curiously, incubating the protein at high concentrations facilitated the assembly of stable ECD tetramers. We do not yet know if the tetrameric form of the protein is biologically relevant or why assembly of the tetramer is slow. However, we did find that once the tetramer formed the protein oligomerized no further, revealing that this is a discrete and defined oligomerization end point. Moreover, upon dilution, the assembled tetramer did not dissociate back into monomers or dimers, suggesting that this may be the mature form of the protein. We hypothesize that the slow rate of assembly may be a consequence of the examining the protein in free solution and that oligomerization of the full-length protein, on the surface of a cell membrane, could be considerably faster. It is also possible that oligomerization may be controlled as a way of regulating transport activity. Consistent with this idea, the full length *C. elegans* Sid-1 functions as an oligomer [5]. Indeed, many transport channels function as symmetric homooligomers (e.g. glutamate transporters [27]; cyclic nucleotide-gated ion channels; [28] aquaporins [29]; potassium channels [30]). Considering the stability of the ECD tetramer it is also conceivable ECD oligomerization could drive formation of the oligomeric Sid-1 pore.

We found that the SidT1 ECD forms a tetrameric, puck-shaped molecule (Figure 4A). Although we could not distinguish between P4 and P222 symmetries using SAXS data, we are inclined to favor the P4 model, as cyclic symmetry is a common feature in membrane proteins with sidedness and/or directionality with respect to the cellular membrane [31]. Because the ECD is the major extracellular portion of Sid-1 it is likely to play a role in substrate recognition, or pore gating, or both. Consistent with this notion, several alleles encoding point mutations that impair import have been identified in the ECD of *C. elegans* Sid-1 [1], [32]. We speculate that the puck-shaped Sid-1 ECD may sit in the aqueous environment atop the transmembrane domains, possibly controlling access to the channel. Interestingly, between the Sid-1 homologs found throughout metazoa, the ECD is the most divergent region of the protein, suggesting that different animal species may have evolved the ECD to confer different functional properties. However, alignment of ECD sequences from several representative Sid-1 homologs revealed a common pattern of predicted secondary structure elements. The secondary structure is predicted to be predominately ß-strands with two large disordered regions that are variable in sequence between Sid-1 homologs (Figure S1). Indeed, limited proteolysis of the *C. elegans* Sid-1 ECD revealed a protease sensitive site likely in variable region 1 (Figure S3B and S4C). The combined observations suggest that the ß-rich ECD fold is conserved among Sid-1 homologs, and thus the biophysical features of the SidT1 ECD reported here may be common to Sid-1 homologs throughout metazoa.

## Materials and Methods

### Plasmid Construction

The expression plasmid, pFB-GP64, was constructed by PCR-amplifying the GP64 promoter and signal sequence from pBAC6 (Novagen), and subcloning into pFastBac HTA (Invitrogen), (see Table S2 for primer sequences and details). The coding region of SidT1 ECD was amplified from a cDNA clone (IMAGE 40125773, Open Biosystems) of full-length SidT1 and cloned as an SfoI-XhoI fragment into the pFB-GP64 plasmid. The resulting plasmid (SidT1-ECD/pFB-GP64) was used in the Bac-to-Bac system (Invitrogen) to generate baculovirus that induce secretion of the SidT1 ECD from insect cells. The encoded protein bears an N-terminal His_6_-tag and tobacco etch virus (TEV) protease site. The final purified protein, following signal peptidase and TEV cleavages, was composed of residues 19–304 (Entrez Protein accession AAI17223), with an N-terminal glycine appendage from the protease site. Baculovirus encoding human SidT2 and *C. elegans* Sid-1 ECDs were produced in the same way. The SidT2 construct encoded residues 19–292 (taken from AAI14523) and the *C. elegans* construct contained residues 17–313 (from NP_504372). Both recombinant proteins contained an additional N-terminal glycine-alanine dipeptide after cleavage by the TEV protease.

### Protein Expression

*Spodoptera frugiperda* 9 (Sf9) or *Trichoplusia ni* (Tni) cells were obtained from Expression Systems (Woodland, CA) and cultured in ESF 921 medium (Expression Systems) containing 0.5× Antibiotic/Antimycotic solution (Gibco). To produce DNA for generating baculovirus, SidT1-ECD/pFB-GP64 was transformed into DH10-Bac *E. coli* (Invitrogen), and bacmid DNA was purified from 2 mL overnight LB cultures using alkaline lysis (Qiagen buffers P1, P2 and N3), followed by isopropanol precipitation. Bacmid DNA was transfected into Sf9 cells using Fugene 6 (Roche). Baculovirus was purified from infected cells and debris 7 days post-transfection by centrifugation and filtration. Three successive viral amplifications were performed, such that viral stocks were used to infect increasing cell volumes for shorter periods of time until the stocks reached optimal titer. Optimal titer was determined empirically by observing viral-induced growth arrest and swelling of infected expression host cells. The kinetics of SidT1 ECD protein expression were monitored by anti-His tag Western blot analysis or small scale Ni affinity purification (described below).

### Protein Purification

For small-scale test expressions, 10–20 mL of cell culture medium containing SidT1 ECD was clarified by centrifugation then dialyzed against nickel-resin wash buffer (20 mM sodium phosphate, 0.5 M NaCl, 0.5 mM TCEP, 20 mM imidazole, pH 8). Dialyzed medium was incubated with two hundred microliters of His Select resin (Sigma) for 10 minutes. The resin was pelleted by brief centrifugation and bound protein was eluted into 50 µL of wash buffer containing 250 mM total imidazole.

For large-scale preparations, SidT1 ECD was purified directly from the cell growth medium, following a 20-minute centrifugation step at 6000×g to remove cells and debris. Clarified medium was loaded onto a 20 ml column of SP Fast Flow (GE Healthcare Life Sciences), pre-equilibrated in 50 mM sodium phosphate, pH 6.5, 25 mM NaCl and 0.5 mM TCEP, and was washed with the same buffer until UV absorbance reached a constant baseline. SidT1 was eluted from the column in a step elution with buffer containing 50 mM sodium phosphate, pH 8, 0.5 M NaCl and 0.5 mM TCEP. SidT1 ECD-containing fractions (∼5 column volumes) were pooled, adjusted to 20 mM imidazole using a 3 M stock (pH 8), and directly applied to up to 5 mL of His-Select Nickel resin (Sigma) equilibrated in nickel-resin wash buffer. After a 60-minute incubation with the resin, SidT1 ECD was washed extensively with the same buffer in batch centrifugation format. The protein was eluted with 3 column volumes of nickel-column elution buffer (20 mM sodium phosphate, 0.5 M NaCl, 0.5 mM TCEP, 250 mM imidazole, pH 8). The TEV protease was added to the eluate and the mixture was dialyzed against nickel-column wash buffer overnight. The TEV-treated, dialyzed sample was passed through a 1 or 5 mL His-trap column (GE Healthcare Life Sciences) and then concentrated using an Amicon Ultra centrifugal filter (Amicon). Concentrated samples were allowed to equilibrate up to several hours on ice and then applied a Superdex 200 10/300 GL column (GE Healthcare Life Sciences) equilibrated in 20 mM HEPES, pH 8, 100 mM NaCl, 0.5 mM TCEP. The eluted protein was concentrated as above and stored at −80°C in 100 µL aliquots at 10 mg/mL, or used immediately. All purification steps were performed at 4°C.

### Protein analysis

Protein purity was assessed by SDS PAGE using Coomassie Staining (Denville Blue) or by Western Blotting using an alkaline-phosphatase conjugated anti-His_6_-tag antibody (Sigma) and colorimetric detection in a solution containing 100 mM Tris, pH 9.6, 100 µg/mL nitro blue tetrazolium, 4 mM MgCl_2_ and 50 µg/mL 5-bromo-4-chloro-3-indolyl-phosphate. MALDI-TOF mass spectrometry analyses were performed at the Scripps Research Institute Center for Metabolomics and Mass Spectrometry. Deglycosylation was done on an analytical scale using PNGaseF from New England Biolabs, or on a preparative scale using purified recombinant PNGaseF (a gift from Dr. Raymond Stevens' lab). The optimal enzyme concentration was determined empirically and was generally performed in nickel-column wash buffer. Native molecular weights were estimated on a Superdex 200 10/30 size exclusion column that was calibrated using an HMW Calibration Kit (GE Healthcare Life Sciences).

Limited proteolysis of *C. elegans* Sid-1 ECD was performed as follows: Two microliters of a dilute trypsin solution (10 µg/mL trypsin in 25 mM Tris, pH 7.5, 150 mM NaCl) was added to to 2 µg of purified *C. elegans* Sid-1 ECD in a total volume of 15 µL. The reaction proceeded for about 3 minutes at room temperature and was stopped by the addition of SDS-PAGE loading buffer. The sample was flash frozen in liquid nitrogen and stored frozen until analysis by SDS PAGE.

### Electron Microscopy

A purified sample of SidT1 extracellular domain was adhered onto a carbon-coated C-flat grid and negatively-stained using 2% uranyl acetate. Micrographs were taken on a FEI Morgagni microscope at 80 kV on a 2 k×2 k CCD camera. Particles appeared well dispersed with an approximate size of 10 nm.

### Small Angle X-ray scattering

Small angle X-ray scattering (SAXS) data were collected on 20 µL samples of purified SidT1 ECD (not treated with glycosidase) at 2.5 mg/mL in phosphate buffered saline containing 0.5 mM TCEP, at 16°C, using a MAR CCD 165 detector on the SIBYLS beamline 12.3.1 at the Advanced Light Source. The x-ray wavelength (λ) was 1 Å, and the sample-to-detector distance was 1.5 m, corresponding to a scattering vector *q* (*q* = 4π sin θ/λ, where 2θ is the scattering angle) range of 0.01 to 0.32 Å^−1^. Exposures of 0.5, 1 and 6 seconds were taken for both the sample and a buffer blank. Scattering of the buffer blank was subtracted from that of the protein, and exposure data sets were merged with PRIMUS [33] using low exposure data for the low-*q* region and high exposure data for the high-*q* region. PRIMUS was also used to calculate the radius of gyration (*R*_G_) using the Guinier approximation for the low-resolution data (*qR*_G_<1.3). The *P*(*r*) function, *R_g_* (real space approximation) and maximum distance *D*_max_ were calculated with GNOM [34]. Using the data range of 0.028 to 0.29 Å^−1^, GASBOR [35] was used to generate 10 independent 3D models in several different symmetry groups. The independent models were aligned and averaged using DAMAVER [36]. For P3 modeling, 1 of the 10 models was rejected by DAMAVER and not used in subsequent averaging. Chi squared values reported are the mean of the independent runs. Normalized spatial discrepancy (NSD) values are reported as the mean value with variability for each symmetry group (Table S1). SAXS data were deposited in the Bioisis database (code SIDT1P, www.bioisis.net).

### Sequence alignment and secondary structure prediction

Sid-1 homolog ECD sequences were aligned using the ClustalW2 server (http://www.ebi.ac.uk/Tools/msa/clustalw2/) [37], with minor manual adjustments. Secondary structure was predicted using the Phyre server (http://www.sbg.bio.ic.ac.uk/~phyre/) [38]. Secondary structural elements with confidence scores of 5 and higher (on a scale of 1–10) were mapped on the sequence alignment.

## Supporting Information

Figure S1 **Secondary structure prediction suggest ECDs of Sid-1 homologs share a conserved fold.** Aligned Sid-1 ECD sequences display conservation of predicted secondary structural elements. Sid-1 ECDs are predicted to be ß -strand rich. All homologs examined have a conserved cysteine in the beginning of ß11, and most homologs (aside from *Caenorhabditis* orthologs) share a second pair of conserved cysteines in the variable 1 and ß11/ß12 linker regions. Predicted sites of N-linked glycosylation are not as well conserved, although some N-enriched regions are evident. Sequences examined include *Homo sapiens* SidT1 (Genbank AAI17223.1); *Xenopus (Silurana) tropicalis* SidT1 (NCBI XP_002941891.1); *Homo sapiens* SidT2 (Genbank AAI14523.1); *Siniperca chuatsi* SidT2 (Genbank ADG29120.1); *Tribolium castaneum* Sid-1 related (Genbank EFA10693.1); *Apis mellifera* SidT1 (NCBI XP_395167.4); *Caenorhabditis elegans* Sid-1(NCBI NP_504372.2); *Caenorhabditis remanei* Sid-1 (NCBI XP_003113953.1); *Strongylocentrotus purpuratus* SidT2 (NCBI XP_001176487.1). Interestingly, the sea urchin Sid-1 homolog appears circularly permuted, with the predicted ECD appearing in the middle of the primary sequence.
(TIF)

Figure S2 **Glycosidase-treated SidT1 ECD maintains its oligomeric state.** PNGaseF-treatment of the SidT1 ECD tetramer increases gel filtration elution volume only very slightly, indicating that the quaternary structure remains intact. Left axis corresponds to absorbance of untreated protein, and right axis corresponds to the PNGaseF-treated sample.
(TIF)

Figure S3 **Expression and Purification of Sid-1 homolog ECDs.** **A**, Western blot against the His_6_ purification tag reveals expression of recombinant human or *C. elegans* Sid-1 ECDs in growth medium of infected Sf9 cells. Recombinant ECDs of human SidT1 (T1), SidT2 (T2), and *C. elegans* Sid-1 (C) are labeled. **B**, SDS PAGE of Sid-1 ECD proteins after cation exchange chromatography. **C**, SDS PAGE of a purified SidT2 ECD sample after cation exchange, nickel affinity and gel filtration purification steps.
(TIF)

Figure S4 **Characterization of recombinant** ***C. elegans*** **Sid-1 ECD.** **A**, PNGaseF treatment of the *C. elegans* Sid-1 ECD results in increased mobility of the protein sample in SDS-PAGE. **B**, Limited proteolysis of the protein results in the release of two lower molecular weight species. **C**, Schematic of the ECD, with respect to the full-length protein, and depiction of the likely trypsin sensitive sites (red scissors). Seven predicted N-linked glycan sites (purple stars) are shown in the cartoon. Molecular weights are theoretical estimates based on the likely trypsin cleavage sites, and do not account for glycosylation.
(TIF)

Figure S5 ***Ab Initio*** **3D Models of SidT1 ECD in Different Spacegroups.** P3, P4, P222 and P5 symmetries were imposed in modeling runs using GASBOR. Models shown are the averages of 10 independent runs (except for the P3 models, in which 9 are averaged). Chi-squared and normalized spatial discrepancy (NSD) values are indicated.
(TIF)

Table S1 **Data Summary.** Calculated and derived values are listed for biophysical measurements, basic SAXS parameters and 3D modeling runs.
(TIF)

Table S2 **Primers for constructing the pFB-GP64 plasmid.** pFBHTA-NdeI-QC-For and -Rev are site directed mutagenesis primers used to introduce a unique *Nhe*I site in pFastBac HT A. PCR amplification of the GP64 promoter from pBac6 used NdeI-GP64 and GP64-SfoI-Rev for directional cloning into the *Nhe*I-modified pFastBac HT A, using *Nhe*I and *Sfo*I restriction sites. This strategy removes the His_6_ tag from the original plasmid, allowing constructs of interest to be cloned as fusion proteins to the GP64 secretion sequence, using any of the original multiple cloning sites downstream of the *Sfo*I site in pFastBac HT A.
(TIF)
