# Supplementary figures and images for: Preparation and Characterization of the Extracellular Domain of Human Sid-1
Source: PLoS One. 2012 Apr 11;7(4):e33607. doi: 10.1371/journal.pone.0033607 (PMC3324469; doi:10.1371/journal.pone.0033607)

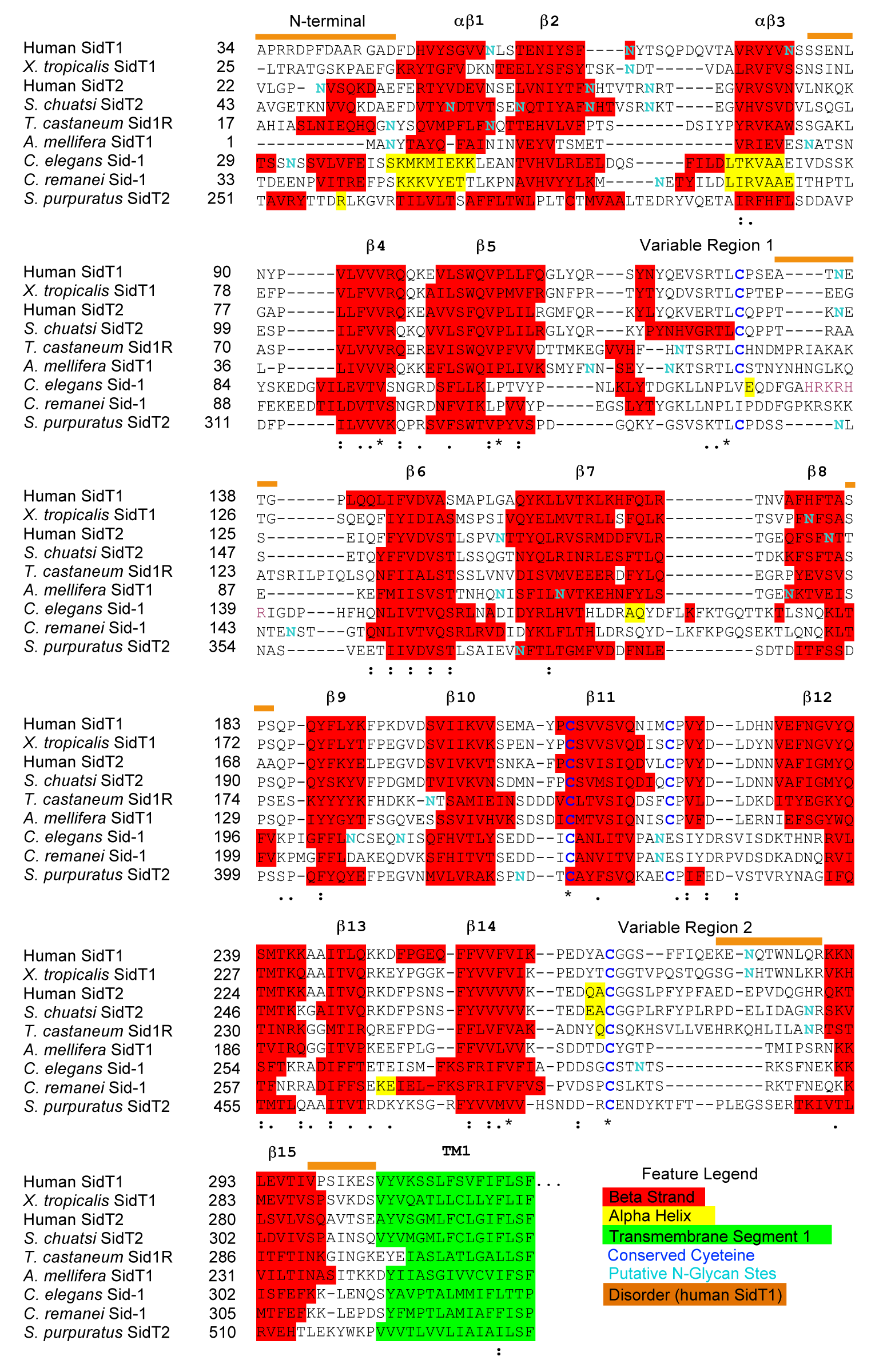

Supplement: Figure S1 — Secondary structure prediction suggest ECDs of Sid-1 homologs share a conserved fold. Aligned Sid-1 ECD sequences display conservation of predicted secondary structural elements. Sid-1 ECDs are predicted to be ß -strand rich. All homologs examined have a conserved cysteine in the beginning of ß11, and most homologs (aside from Caenorhabditis orthologs) share a second pair of conserved cysteines in the variable 1 and ß11/ß12 linker regions. Predicted sites of N-linked glycosylation are not as well conserved, although some N-enriched regions are evident. Sequences examined include Homo sapiens SidT1 (Genbank AAI17223.1); Xenopus (Silurana) tropicalis SidT1 (NCBI XP_002941891.1); Homo sapiens SidT2 (Genbank AAI14523.1); Siniperca chuatsi SidT2 (Genbank ADG29120.1); Tribolium castaneum Sid-1 related (Genbank EFA10693.1); Apis mellifera SidT1 (NCBI XP_395167.4); Caenorhabditis elegans Sid-1(NCBI NP_504372.2); Caenorhabditis remanei Sid-1 (NCBI XP_003113953.1); Strongylocentrotus purpuratus SidT2 (NCBI XP_001176487.1). Interestingly, the sea urchin Sid-1 homolog appears circularly permuted, with the predicted ECD appearing in the middle of the primary sequence. (TIF) [file pone.0033607.s001.tif]

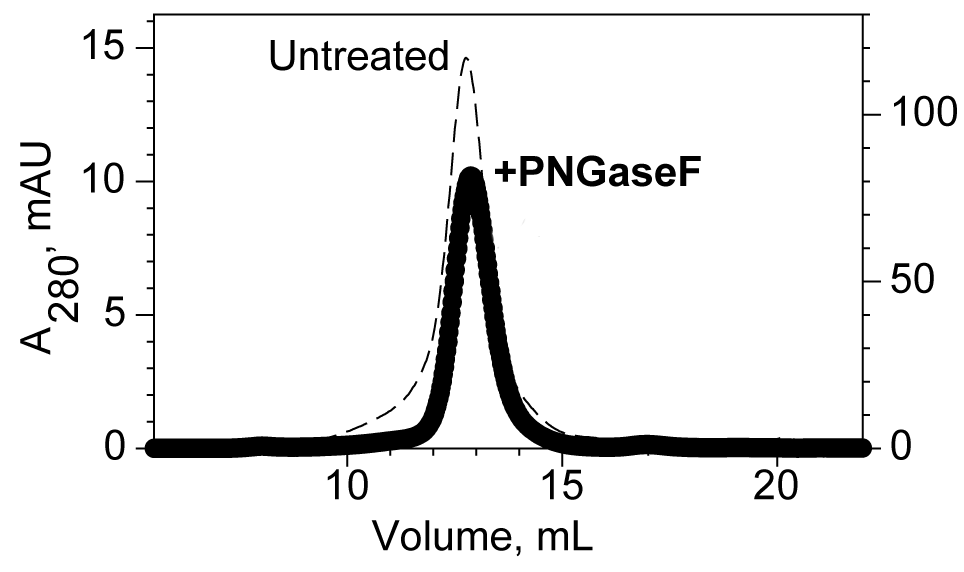

Supplement: Figure S2 — Glycosidase-treated SidT1 ECD maintains its oligomeric state. PNGaseF-treatment of the SidT1 ECD tetramer increases gel filtration elution volume only very slightly, indicating that the quaternary structure remains intact. Left axis corresponds to absorbance of untreated protein, and right axis corresponds to the PNGaseF-treated sample. (TIF) [file pone.0033607.s002.tif]

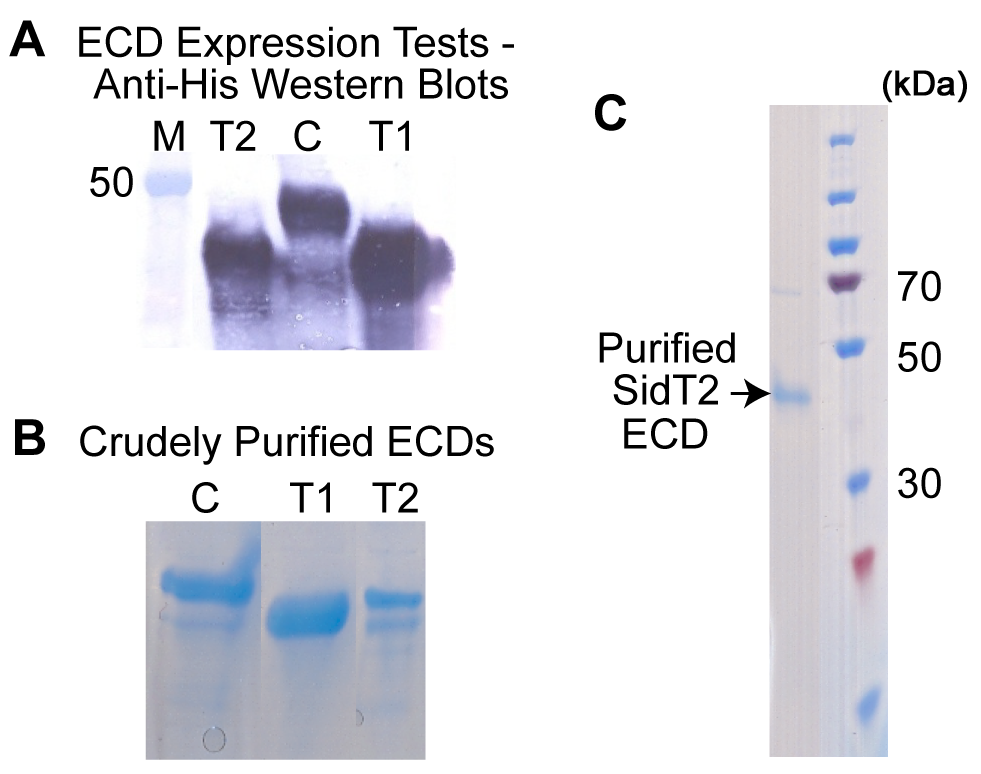

Supplement: Figure S3 — Expression and Purification of Sid-1 homolog ECDs. A, Western blot against the His6 purification tag reveals expression of recombinant human or C. elegans Sid-1 ECDs in growth medium of infected Sf9 cells. Recombinant ECDs of human SidT1 (T1), SidT2 (T2), and C. elegans Sid-1 (C) are labeled. B, SDS PAGE of Sid-1 ECD proteins after cation exchange chromatography. C, SDS PAGE of a purified SidT2 ECD sample after cation exchange, nickel affinity and gel filtration purification steps. (TIF) [file pone.0033607.s003.tif]

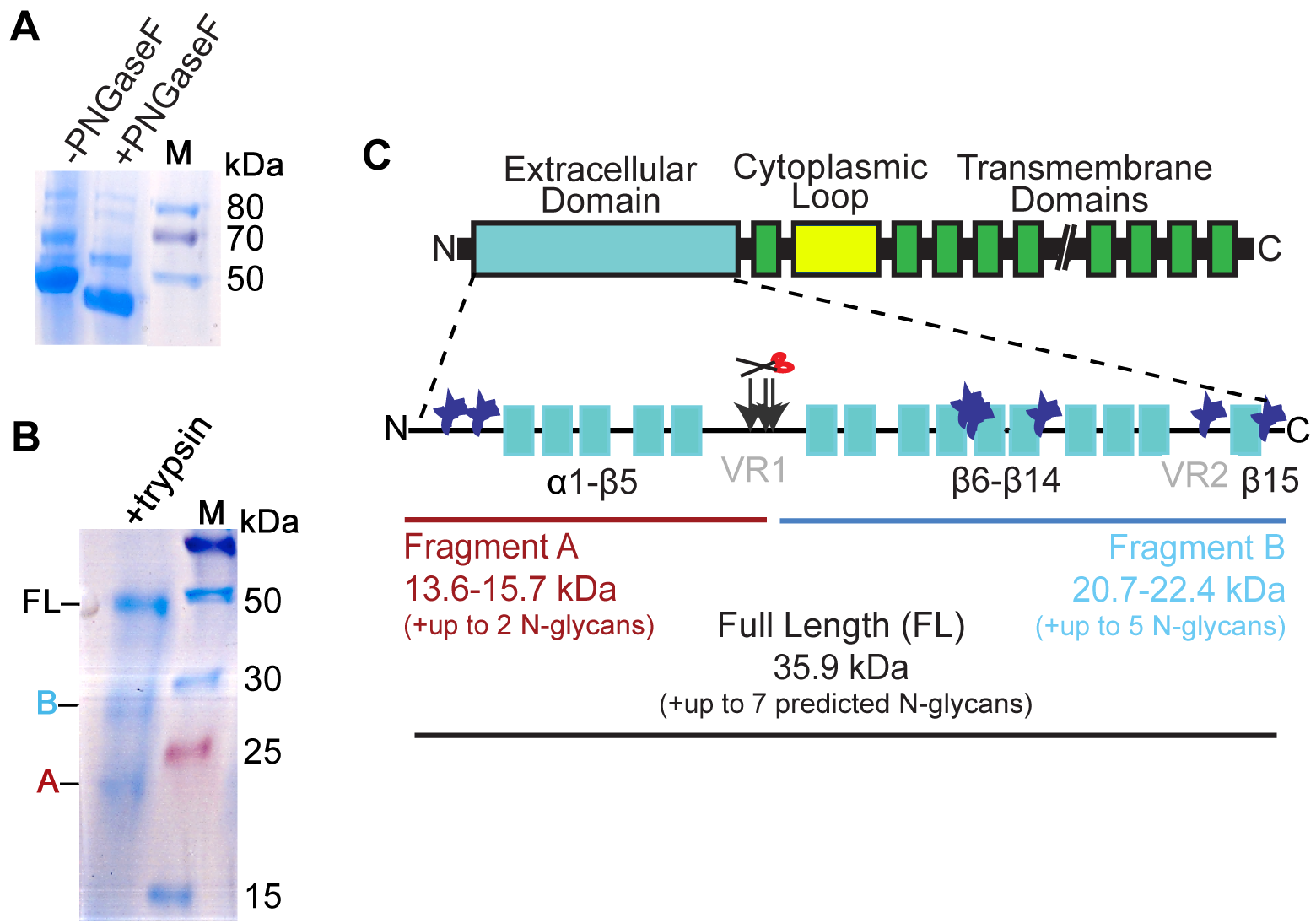

Supplement: Figure S4 — Characterization of recombinant C. elegans Sid-1 ECD. A, PNGaseF treatment of the C. elegans Sid-1 ECD results in increased mobility of the protein sample in SDS-PAGE. B, Limited proteolysis of the protein results in the release of two lower molecular weight species. C, Schematic of the ECD, with respect to the full-length protein, and depiction of the likely trypsin sensitive sites (red scissors). Seven predicted N-linked glycan sites (purple stars) are shown in the cartoon. Molecular weights are theoretical estimates based on the likely trypsin cleavage sites, and do not account for glycosylation. (TIF) [file pone.0033607.s004.tif]

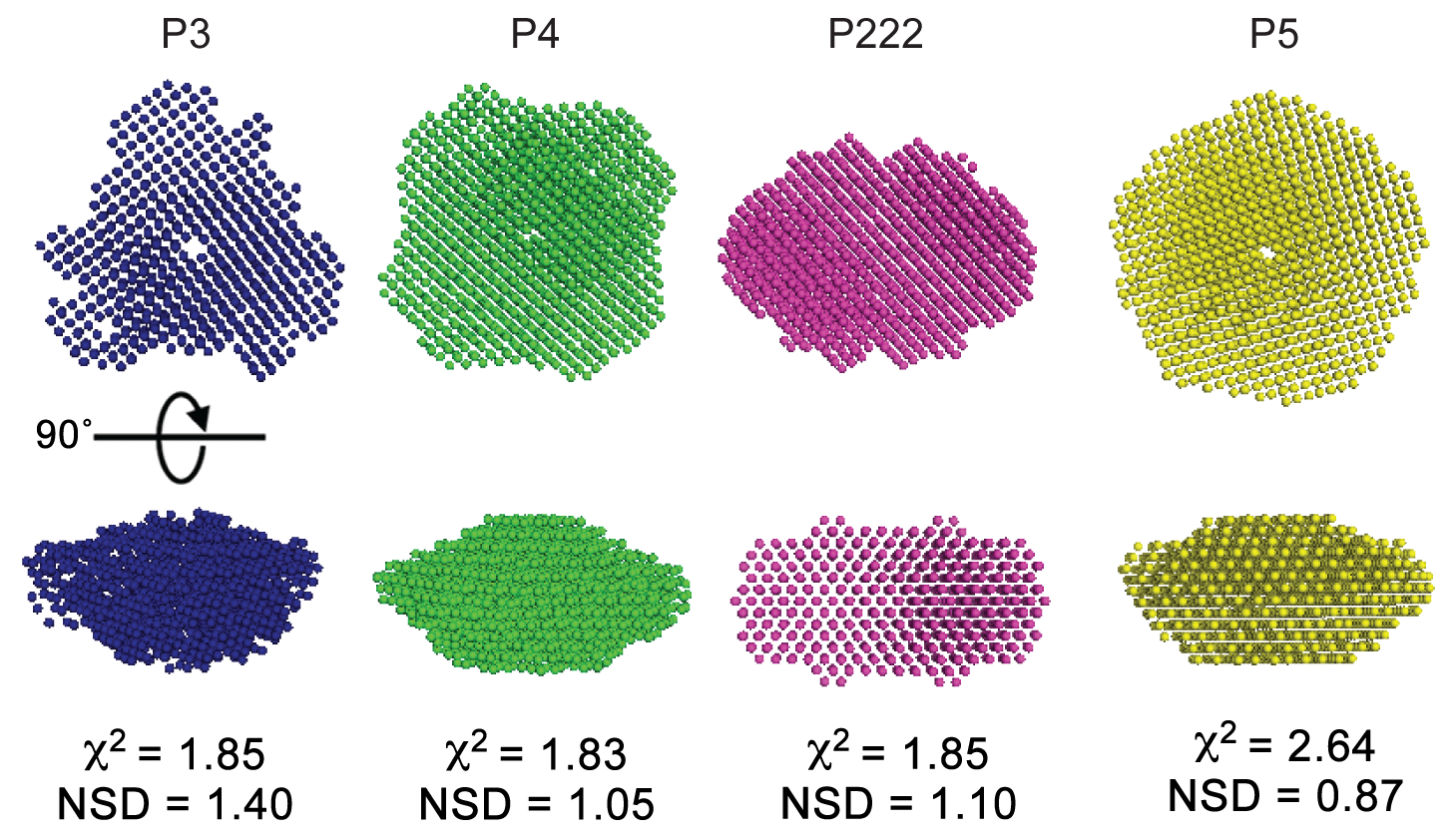

Supplement: Figure S5 — Ab Initio 3D Models of SidT1 ECD in Different Spacegroups. P3, P4, P222 and P5 symmetries were imposed in modeling runs using GASBOR. Models shown are the averages of 10 independent runs (except for the P3 models, in which 9 are averaged). Chi-squared and normalized spatial discrepancy (NSD) values are indicated. (TIF) [file pone.0033607.s005.tif]

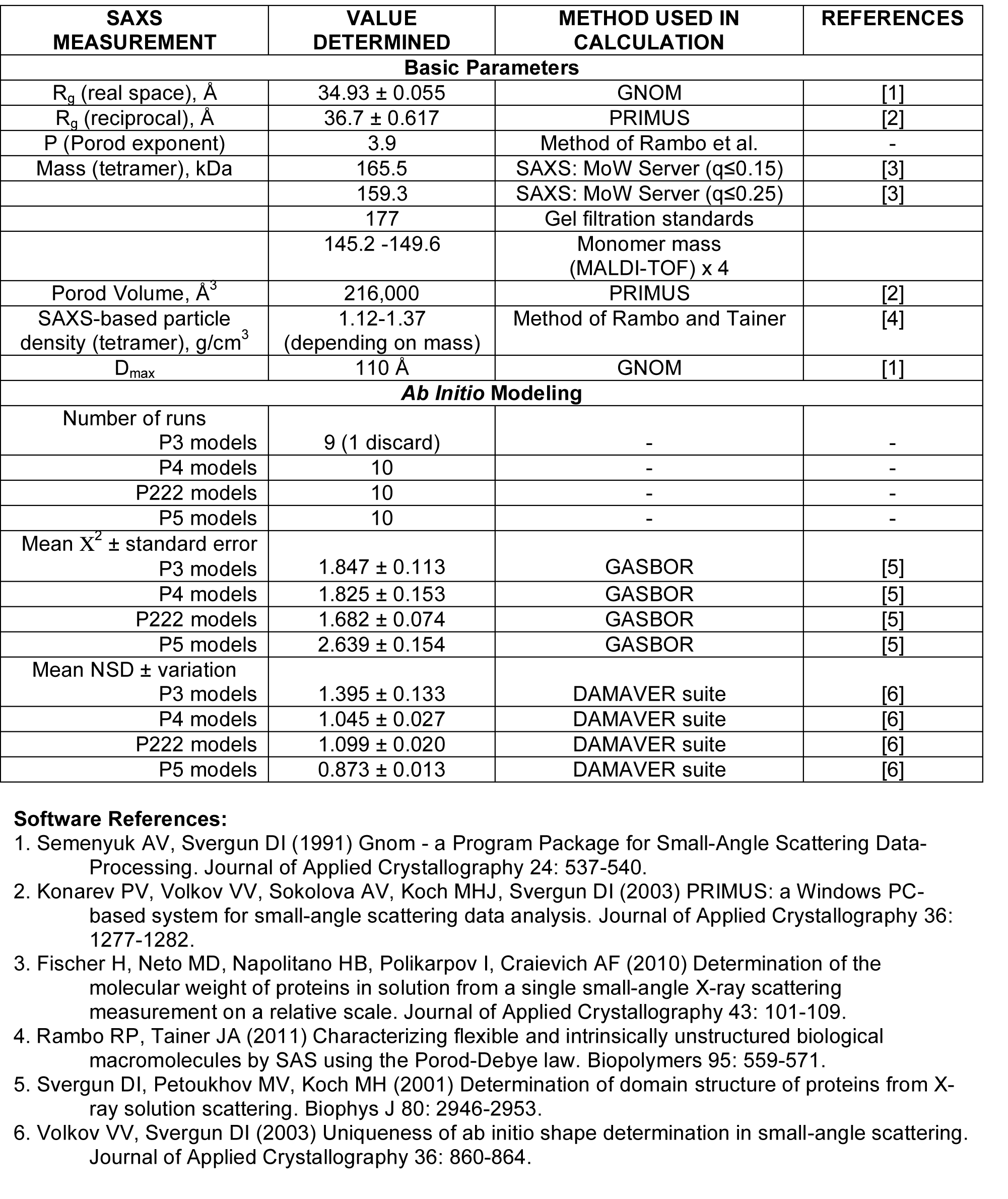

Supplement: Table S1 — Data Summary. Calculated and derived values are listed for biophysical measurements, basic SAXS parameters and 3D modeling runs. (TIF) [file pone.0033607.s006.tif]

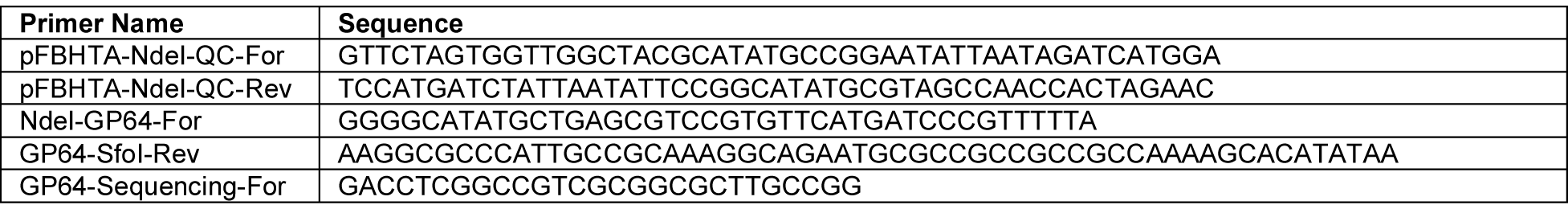

Supplement: Table S2 — Primers for constructing the pFB-GP64 plasmid. pFBHTA-NdeI-QC-For and -Rev are site directed mutagenesis primers used to introduce a unique NheI site in pFastBac HT A. PCR amplification of the GP64 promoter from pBac6 used NdeI-GP64 and GP64-SfoI-Rev for directional cloning into the NheI-modified pFastBac HT A, using NheI and SfoI restriction sites. This strategy removes the His6 tag from the original plasmid, allowing constructs of interest to be cloned as fusion proteins to the GP64 secretion sequence, using any of the original multiple cloning sites downstream of the SfoI site in pFastBac HT A. (TIF) [file pone.0033607.s007.tif]
